# Reclassification calibration test for censored survival data: performance and comparison to goodness-of-fit criteria

**DOI:** 10.1186/s41512-018-0034-5

**Published:** 2018-07-26

**Authors:** Olga V. Demler, Nina P. Paynter, Nancy R. Cook

**Affiliations:** Division of Preventive Medicine, Brigham and Women’s Hospital, 900 Commonwealth Ave, Brookline, MA 02115 USA

**Keywords:** Risk reclassification, Calibration, Goodness-of-fit test, Survival analysis, Hosmer-Lemeshow, Grønnesby-Borgan

## Abstract

**Background:**

The risk reclassification table assesses clinical performance of a biomarker in terms of movements across relevant risk categories. The Reclassification- Calibration (RC) statistic has been developed for binary outcomes, but its performance for survival data with moderate to high censoring rates has not been evaluated.

**Methods:**

We develop an RC statistic for survival data with higher censoring rates using the Greenwood-Nam-D’Agostino approach (RC-GND). We examine its performance characteristics and compare its performance and utility to the Hosmer-Lemeshow goodness-of-fit test under various assumptions about the censoring rate and the shape of the baseline hazard.

**Results:**

The RC-GND test was robust to high (up to 50%) censoring rates and did not exceed the targeted 5% Type I error in a variety of simulated scenarios. It achieved 80% power to detect better calibration with respect to clinical categories when an important predictor with a hazard ratio of at least 1.7 to 2.2 was added to the model, while the Hosmer-Lemeshow goodness-of-fit (gof) test had power of 5% in this scenario.

**Conclusions:**

The RC-GND test should be used to test the improvement in calibration with respect to clinically relevant risk strata. When an important predictor is omitted, the Hosmer-Lemeshow goodness-of-fit test is usually not significant, while the RC-GND test is sensitive to such an omission.

**Electronic supplementary material:**

The online version of this article (10.1186/s41512-018-0034-5) contains supplementary material, which is available to authorized users.

## Background

Risk prediction is viewed as an important part of clinical decision making. For cardiovascular disease and breast cancer, the development of a new risk prediction model has led to changes in practice guidelines. For example, the American College of Cardiology/American Heart Association (ACC/AHA) 10-year cardiovascular disease (CVD) risk model developed from pooled cohorts is currently used in cardiovascular medicine [1], and the Gail model of 5-year risk of developing breast cancer is an application of risk prediction models in cancer [2].

Risk prediction model development typically follows the following steps [3]. First, biomarkers for the new model are selected usually based on significance of their regression coefficients (from Wald or likelihood ratio tests). Once association is established, model performance is assessed usually in terms of its discrimination (measured by area under the receiver operating characteristic curve (AUC) and net reclassification improvement (NRI) among others) and calibration (i.e., Hosmer-Lemeshow goodness-of-fit test, calibration slope, etc.).

Given that absolute risk often defines the treatment prescribed, it is very important to ensure that the model is well calibrated (or that predicted risk is close to its true value). A model can perform well based on tests of association or measures of discrimination but have poor calibration characteristics. Van Calster et al. introduced a four-level hierarchy of risk calibration [4]: mean (or calibration in the large, i.e., the average of predicted risk is the same as observed average risk), weak (or calibration intercept and slope equal zero and one respectively [5, 6]), moderate (or calibration in subgroups of risk assessed with calibration plot, Hosmer-Lemeshow test [7]), and strong (or calibration in various covariate patterns). When the true biological model is known (in terms of inclusion of all important predictor variables in correct functional form), then maximum likelihood estimation of model parameters will produce an asymptotically strongly calibrated model. In practice, the true model is never known and we can only hope that the given model is close to the true model, produces reasonable approximation of risk estimates, and performs well in important subgroups. When the true model is unknown, maximum likelihood estimation guarantees only calibration in the large. It does not guarantee for example a good discrimination and calibration in subgroups as noted by Zhou et al. [8].

Calibration in subgroups defined by risk strata is important for assessing the impact of a new predictor on medical decision-making process. Risk stratification is routinely used in clinical practice. Since in most clinical areas, physicians have a choice of relatively few treatment options, risk is often stratified and different treatments are prescribed for different risk strata. For example, the most recent ACC/AHA cholesterol guidelines recommend that treatment be guided by overall cardiovascular risk. Specifically, in primary prevention, for those aged 40–75 years, 10-year risk should be assessed, and if it is above 7.5%, then consideration of moderate to high intensity statin therapy is recommended along with patient discussion [1]. The American Society of Clinical Oncology recommends consideration of tamoxifen/raloxifene/exemestane therapy as an option to reduce the risk of invasive breast cancer if 5-year breast cancer risk is at least 1.66% in premenopausal women aged 35 years and above [9]. The National Osteoporosis Foundation chose a 10-year hip fracture probability of 3% as an osteoporosis intervention treatment threshold [10]. These examples show that risk stratification is an important component of the medical decision-making process.

When risk stratification is of interest, a relevant question is how adding a given biomarker to a risk model affects clinical decision making [11]. Does it result in more (or less) intensive treatment assignment? A biomarker resulting in many very small adjustments to absolute risk might lead to a significant test of association but in practice may not affect ranges of clinical interest and therefore will have very small effect on clinical decision making. On the other hand, if many individuals change *risk strata*, this may translate into differences in monitoring or treatment. As we have mentioned earlier, maximum likelihood methods do not guarantee good calibration in the subgroups including those defined by risk strata. The risk reclassification table [12] is one of the tools that can be used to assess clinical performance in terms of movements across relevant risk strata. Besides assessing discrimination, it can be used to assess calibration within subgroups defined by these risk strata. While it was originally developed for binary outcome data, it has been used in low-censoring survival data [13]. The performance of the reclassification calibration (RC) statistic for moderate to high censoring rates has not been evaluated. Below, we provide an adaptation of the statistic to the survival setting and explore its properties.

While the extent of change in risk strata is important clinically, whether these changes lead to better model calibration must be considered. A reclassification table is an informative way to display these data. The risk reclassification table was introduced by Cook et al. [12] and is defined in the following section.

## Methods

### Definition of the risk reclassification table

In Table 1, we use the reclassification table generated from the Women’s Health Study (WHS) data to compare models with and without current smoking (left) and the uninformative biomarker (right) predicting hard CVD events. The WHS is a large-scale nationwide 10-year cohort study of women, which commenced in 1992 [14]. Data include 27,464 women with a median age at baseline of 52 years with an age range of 38 to 89 years. The median follow-up is 10.2 years up through March 2004. A total of 600 women developed hard CVD by 10 years of follow-up, and 36.6% of women were censored prior to year 10, most of the censoring occurring after year 8 (with only 1.4% censored prior to year 8). The 2013 ACC/AHA guidelines recommend that “initiation of moderate-intensity statin therapy be considered for patients with predicted 10-year ‘hard’ ASCVD risk of 5.0% to < 7.5%” [1]. We used these thresholds to define risk prediction categories in RC tables presented in Table 1. The left column and the top row in each table define the risk categories produced by the reduced and by the full model correspondingly.

**Table 1:** Reclassification table for informative and uninformative predictors in Women’s Health Study (*N* = 27,464)

Risk category		0–5%	5–7.5%	7.5%+		Risk category		0–5%	5–7.5%	7.5%+
0–5%	ev	289	21	27		0–5%	ev	328	9	0
	ne	23,843	346	217			ne	24,313	93	0
5–7.5%	ev	26	48	12		5–7.5%	ev	7	73	6
	ne	471	688	130			ne	81	1162	46
7.5%+	ev	0	18	145		7.5%+	ev	0	3	160
	ne	0	263	920			ne	0	47	1136

Left: rows—categories defined by the reduced model (controlling for age, total cholesterol, HDL cholesterol, systolic blood pressure and diabetes) and columns—categories defined by the reduced model + current smoking

Right: rows—categories defined by the reduced model and columns—categories defined by the reduced model + uninformative predictor

On the diagonal is the number of people (non-events and events) who do not change categories. Based on the left table, inclusion of current smoking resulted in transition of 21 and 346 non-events from the lowest to the middle risk category, while 23,843 and 289 events remained in the lowest risk category. Addition of the non-informative biomarker resulted in the reclassification table with very few observations in the off-diagonal cells.

Risk categories are sometimes used in clinical decision making to assign treatment as is the case in cardiovascular disease and breast cancer. When choosing between two risk prediction models, we then should consider groups of patients who will be affected by the switch to a new risk prediction model and evaluate whether the proposed reclassification is beneficial. We can ask the question whether the new risk categorization is closer to the actual risk, and we can use the RC test to test this hypothesis. While reclassifications can improve the fit, movement due to chance must also be accounted for. To evaluate the quality of reclassification, a reclassification calibration statistic was introduced [12, 13, 15]. It evaluates similarity between observed and expected counts in each cell of the reclassification table. The test of the RC statistic in logistic regression and in the survival setting with low censoring rates has the following form [15]:

1 $$ {\chi}_{\mathrm{RC}}^2={\sum}_{g=1}^G\frac{{\left[{O}_g-{n}_g{\overline{p}}_g\right]}^2}{n_g{\overline{p}}_g\left(1-{\overline{p}}_g\right)} $$

where *O*_*g*_ is observed number of events in the *g*th cell, ${\overline{p}}_g$ is the average of predicted probabilities for the model in question, *n*_*g*_ is the number of observations in the *g*th cell, and *G* is the number of cells in the RC table. The test is similar to the Hosmer-Lemeshow test using categories defined by the cross-tabulation of risk strata from the two models. Its performance characteristics have been described [13], and the power and Type I error found to be appropriate in this setting. In this paper, we developed a robust test of RC statistic in survival setting.

The reclassification table by construction compares the performance of two models; therefore, there are two ways to calculate the expected counts of events in each cell in (1). One is based on predicted probabilities from the full, and the other is based on the reduced model’s predicted probabilities. Technically, the two RC tests can result in four possible testing combinations, as illustrated in Table 2.

**Table 2:** The implications of RC testing

		RC test based on predicted probabilities from the new model	
		Statistically significant	Not significant
RC test based on predicted probabilities from the old model	Statistically significant	A. Both new and old models are miscalibrated or use incorrect functional form	B. New model provides improved calibration across risk classifications
	Not significant	C. New model is miscalibrated or uses incorrect functional form. Old model is preferable.	D. Reclassification is not choosing between models.

Comparison of RC test with expected counts calculated from the old model with RC test with expected counts calculated from the new model

Typically, when a new important predictor is added to a model, or a fuller model is used, the RC test for the old model indicates significant deviation from the observed rates, while the new model matches the observed rates more closely, as in Table 2 (cell B). More rarely, when the new model is significant (Table 2 (cells A and C)), then the new model is miscalibrated or uses an incorrect functional form. If both models show significant deviations (Table 2 (cell A)), both are miscalibrated. If the models are not nested, it is possible that each model contains unique predictors that are important to prediction. If there is little reclassification, both RC statistics may be non-significant. In this case, either model could be used or other criteria, such as model simplicity or cost, should be used to choose between the models. In practice, we observe mostly the situation described by cells B and D in Table 2.

### Risk reclassification and calibration for survival data

#### Notation

For all *N* observations in the dataset, we assumed that the following data are collected: covariates measured at baseline (*x*_1_,…,*x*_*p*_), event occurrence, and *T* = time of event or administrative censoring (i.e., all observations who did not have an event by the year 10 are censored at *T* = 10). We assume that event times can be right censored and made the usual assumption of independent censoring. We denote *δ* as an event indicator (*δ* = 1, if the event was observed; *δ* = 0, if censored) and we observe *T* = time of event or censoring time, whichever occurs first.

**Fig. 1:**
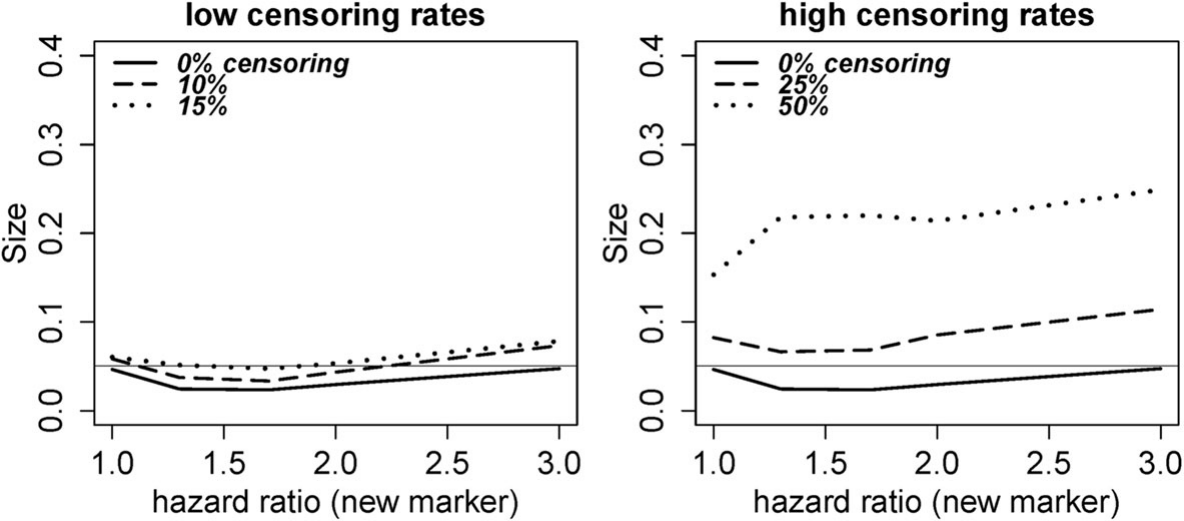
Size of the original RC test (1) for low and high censoring rates. An uninformative new marker is added to a baseline model. Size is calculated as a fraction of significant RC statistics

In order to apply this test to studies with long follow-up and censored observations, we need to extend the test (1) to the survival setting. Cook and Ridker [15] applied the Nam-D’Agostino test [16] to the reclassification table in the survival setting with low censoring rate and suggested estimating the observed proportion *O*_*g*_/*n*_*g*_ using the non-parametric Kaplan-Meier estimator. Expected probabilities in the original formula (1) are replaced with model-based predicted probabilities (i.e. based on Cox model) calculated at a fixed time *t* and averaged for each cell (denoted as ${\overline{p(t)}}_g$) as illustrated in Table 1. In order to test improvement of classification, expected probabilities in each cell are estimated as an average of predicted probabilities from the new model.

2 $$ {\chi}_{\mathrm{RC}}^2(t)={\sum}_{g=1}^G\frac{{\left[{KM}_g-{\overline{p(t)}}_g\right]}^2}{{\overline{p(t)}}_g\left(1-{\overline{p(t)}}_g\right)/{n}_g}{\sim}_{H_0}\kern0.5em {\chi}_{G-1}^2 $$

where *KM*_*g*_ is the observed probability of an event in group *g* estimated using Kaplan-Meier non-parametric estimate. In Fig. 1, we present results using simulated data to compare the size of this version of the RC test for survival with low and high censoring rates. In the absence of censoring, the RC test performs well at the targeted 5% significance level, but then quickly deteriorates and becomes too conservative for higher censoring rates. In this paper, we investigate ways of adapting the original RC test to higher censoring rates in the survival setting, discuss their performance under a variety of scenarios, and compare its performance to the Hosmer-Lemeshow test.

To adapt (2) to the survival setting with high censoring rates, we considered two options: the Grønnesby-Borgan (GB) [17, 18] and the Greenwood-Nam-D’Agostino (GND) [19] tests. These two tests extend Hosmer-Lemeshow style goodness-of-fit tests for survival models and both perform well in a variety of settings [19]. Differences in underlying principles behind the two tests lead to different advantages and different limits of applicability.

#### Greenwood-Nam-D’Agostino test

Nam and D’Agostino formulated a test which is also based on the difference between observed and expected number of events, but uses scaled up versions of the observed counts [16]. Their test uses the Kaplan-Meier estimate of the number of events that would occur without censoring. Their test is valid for low-censoring scenarios and has been extended for higher censoring rates in [19]. The new version of this test is called Greenwood-Nam-D’Agostino (GND) test, because it uses the Greenwood variance formula [20] in the denominator. The GND test performs well for higher censoring rates and is defined as

3 $$ {\chi}_{\mathrm{GND}}^2(t)={\sum}_{g=1}^G\frac{{\left[{KM}_g(t)-{\overline{p(t)}}_g\right]}^2}{\mathrm{Var}\left({KM}_g(t)\right)}{\sim}_{H_0}\kern0.5em {\chi}_{G-1}^2 $$

#### Grønnesby-Borgan test

Using martingale theory, Grønnesby and Borgan developed a test of fit for Cox proportional hazards regression models [18]. It is based on the difference between the observed and expected number of events in deciles, but it can be applied to any grouping. Previously [19], we showed that the GND test has comparable or sometimes superior performance to the Grønnesby and Borgan (GB) test. In this paper, we applied the GB and the GND tests to the reclassification table, denoting them RC-GB and RC-GND. We concluded that the GND test is superior; therefore, in this paper, we focused on the RC-GND test. Results related to the performance of the RC-GB test are presented in Additional file 1: Figure S1, and Additional file 2: Figure S2.

The goal of this paper is to extend the RC statistic to the survival setting with higher censoring rates, compare its performance to the Hosmer-Lemeshow goodness-of-fit, and relate it to existing measures of performance of risk prediction models. In the following sections, we compared performance of the two tests in simulations, discuss differences between the reclassification table and HL type approaches, and apply our findings to the practical example.

#### Simulation setup

Samples of size *N* = 1000, 5000, and 10,000 were generated 1000 times. Event times were generated from the Weibull distribution with the shape parameter *α* set to 3.0 for models with increasing baseline hazard and 0.3 for models with the decreasing baseline hazard. The scale parameter of the Weibull distribution was proportional to exponentiated risk score of the data-generating model, i.e., rs = ln(8)*x*_1_ + ln(1.0,1.3,1.7,2.0,3.0)*x*_2_, where *x*_1_*~N*(0,0.5) and *x*_*2*_*~N*(0,0.5). The scale parameter of the Weibull distribution was also calibrated to an 0.1 event incidence rate. Censoring times were uniformly distributed to generate 0, 25, and 50% censoring rates. Cox proportional hazards models were used to fit the data. The RC table was calculated with cutoffs of 5 and 20% for the simulated data.

Two models were compared: the full model with *x*_1_ and *x*_2_ and a reduced model with only one predictor variable *x*_1_. To estimate the size of the proposed tests, probabilities from the full model were used to estimate the expected proportions in (3). In this case, we would expect the RC statistic to be non-significant because the data are generated under the null. To estimate the power of the proposed tests, probabilities from the reduced model were used to estimate the expected proportions in (3). In this case, we would expect the RC statistic to be significant because the data are under the alternative. We evaluate power in other scenarios as well. Simulations were performed using R statistical software [21].

In a reclassification table, off-diagonal elements can be small or even zero. Bias of the Greenwood variance estimator in such small subgroups is negative and can be as high in absolute value as 25% [22]. For these reasons, the GND test deteriorates for small cell sizes. To accommodate this, we used the following collapsing strategy. All cells with less than five events were collapsed with the nearest cell which is closer to the diagonal and the null setting. In this way, we keep all the data and avoid problems with small cells, although we are biasing the test toward the reference model to some degree. If collapsing was performed, then the degrees of freedom of the test should be adjusted accordingly. The collapsing strategy is illustrated in Table 3.

**Table 3:** Collapsing strategy of the reclassification table

			Full model with total cholesterol		
			0–5%	5–7.5%	7.5%+
Without total cholesterol	0–5%	ev	302	15	←1
		ne	24,137	282	←6
	5–7.5%	ev	14	54	19
		ne	215	852	137
	7.5%+	ev	0→	10	167
		ne	3→	141	1105

Cells with less than five events are collapsed with the next cell closer to the diagonal. Columns—categories defined by the full model (controlling for age, total cholesterol, HDL cholesterol, systolic blood pressure, current smoking, and diabetes) and rows—categories defined by the full model without total cholesterol

*ev* number of events, *ne* number of non-events

## Results

### Performance of the GND test for the RC statistic

#### Size

**Fig. 2:**
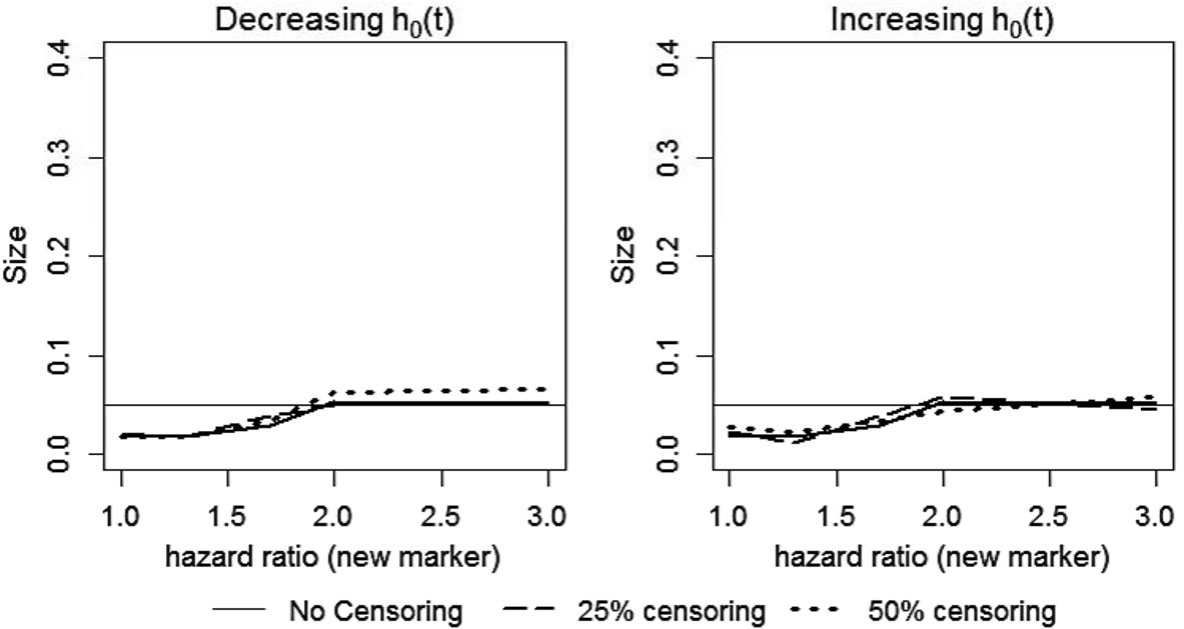
Size of the RC Greenwood-Nam-D’Agostino test (RC-GND) (2) for 0, 25, and 50% censoring rates. Comparing full (*y*~ *x*_1_ + *x*_2_) and reduced (*y*~ *x*_1_) models with decreasing (left) and increasing (right) baseline hazard functions. *N* = 5000, *p* = 0.1, collapse when *ev*_*g*_ < 5

As described in the previous section, we generated a reclassification table for the full model with two predictor variables *x*_1_ and *x*_2_ and a reduced model with only one predictor variable *x*_1_. Full model was used to generate data and to estimate the expected proportions in the RC statistic formula. Detailed explanation of the simulations is presented in the Additional file 3: Table S1. In Fig. 2, we show the size of the RC-GND tests for decreasing (left) and increasing (right) baseline hazards. The RC-GND test is robust to censoring when compared to Fig. 1. In general, the RC-GND test does not exceed targeted Type I error rate (we used 5% significance level in this paper) but can be too conservative when effect size is moderate.

#### Power

To evaluate power, we considered several scenarios, including omission of an important predictor variable, omission of a squared term, and omission of an interaction term. Simulations scenarios are summarized in the Additional file 3: Table S1.

##### Power of RC-GND when omitting an important predictor variable

**Fig. 3:**
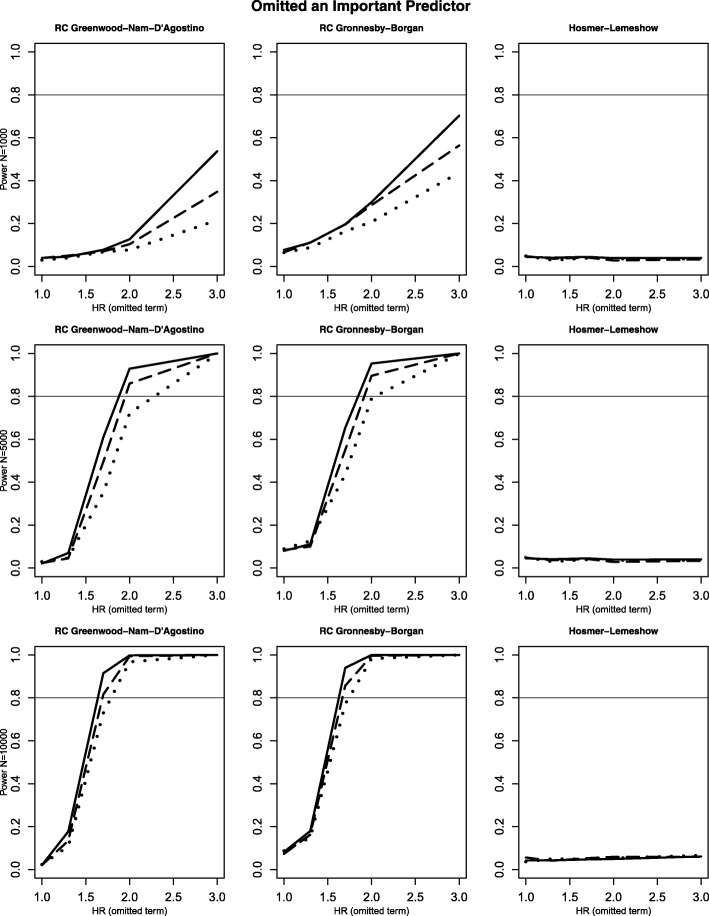
Power when an important predictor is omitted for 0, 25, and 50% censoring rates. The RC statistic was calculated for the full (*y*~ *x*_1_ + *x*_2_) and reduced (*y*~ *x*_1_) models. Data were simulated according to the full model, but the reduced model was used to estimate the expected proportions in the RC statistic formula. Left panel—decreasing baseline hazard, right panel—increasing baseline hazard. *N* = 1000 (top row), 5000 (middle row), 10,000 (bottom row), *p* = 0.1, collapse when *ev*_*g*_ < 5

Data were simulated according to the correct full model, but the reduced model was used to estimate the expected proportions in the RC statistic formula, thus mimicking the situation when an important predictor variable was omitted. Based on Fig. 3, the RC-GND test loses power for hazard ratios less than 2.0 and achieves 80% power for HR > 2.0 and decreasing baseline hazard.

##### Power of RC-GND when omitting squared term

**Fig. 4:**
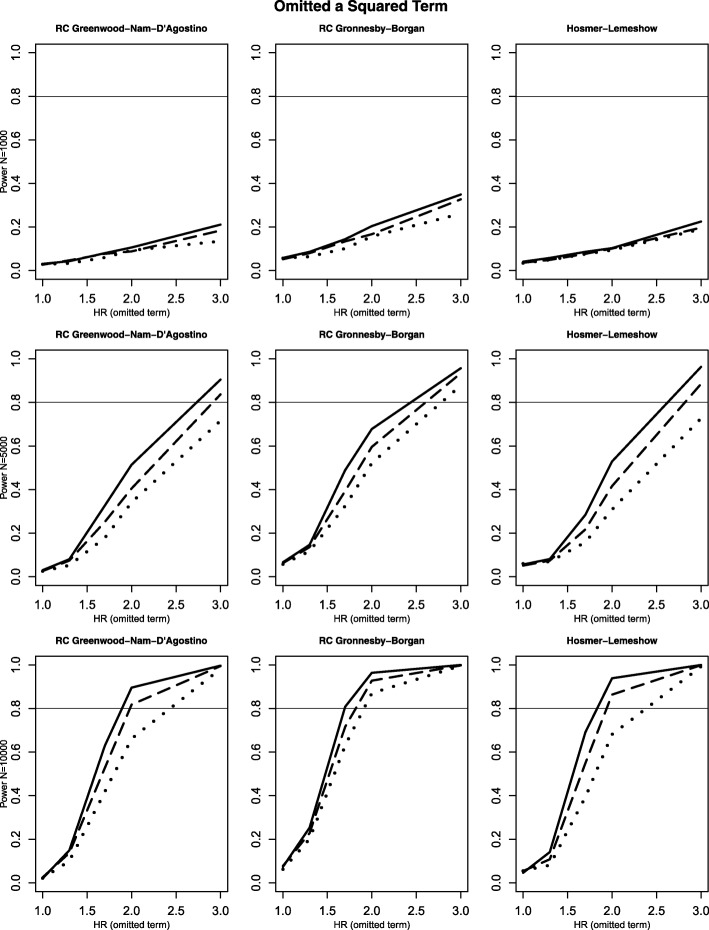
Power when a squared term is omitted for 0, 25, and 50% censoring rates. The RC statistic was calculated for the full (*y*~ *x*_1_ and *x*_1_^2^) and reduced (*y*~ *x*_1_) models. Data were simulated according to the full model, but the reduced model was used to estimate the expected proportions in the RC statistic formula. Left panel—decreasing baseline hazard, right panel—increasing baseline hazard. *N* = 1000 (top row), 5000 (middle row), 10,000 (bottom row), *p* = 0.1, collapse when *ev*_*g*_ < 5

In Fig. 4, we generated survival times according to the model with two predictor variables: *x*_1_ and${x}_1^2$, in the reclassification table and we compared it to the model with only *x*_1_, thus omitting the squared term. RC-GND is robust to censoring for a decreasing baseline hazard (Fig. 4).

##### Power of RC-GND when omitting an interaction term

**Fig. 5:**
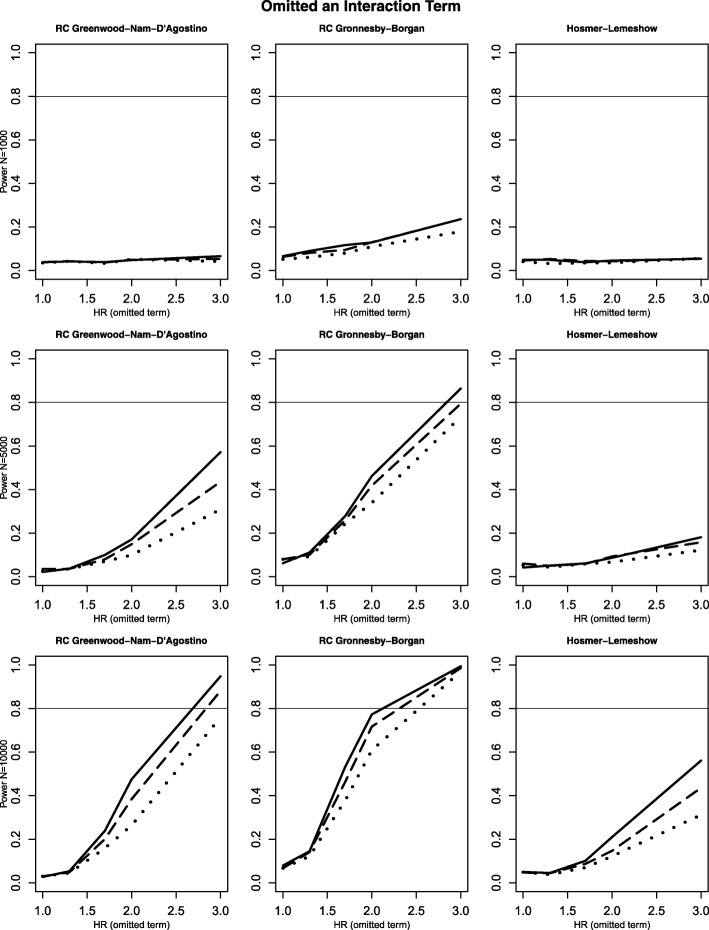
Power when an interaction term is omitted for 0, 25, and 50% censoring rates. The RC and HL statistics were calculated for the following reduced and full models: *y*~ *x*_1_ and *y*~ *x*_1_ + *x*_2_; *y*~ *x*_1_ and *y*~ *x*_1_ + *x*_1_^2^; and *y*~ *x*_1_ + *x*_2_ and *y*~ *x*_1_ + *x*_2_ + *x*_1_ * x_2_. Data were simulated according to the full model, but the reduced model was used to estimate the expected proportions in the RC statistic formula. Left column—power of the RC-GND test, right column—power of the HL gof test. *N* = 1000 (top row), 5000 (middle row), 10,000 (bottom row), *p* = 0.1, collapse when *ev*_*g*_ < 5

Similar results were obtained when omitting an interaction term and are presented in the Fig. 5.

### Connection between the RC statistic, the NRI, and the HL test

#### The RC statistic and net reclassification improvement (NRI)

The NRI is a measure of improvement in predictive model performance [23] which gained popularity in recent years. Its categorical version is defined as the fraction of correct movements across categories among events plus the fraction of correct movements among non-events:

$$ {\mathrm{NRI}}_{\mathrm{cat}}=\frac{\#\mathrm{ca}{{\mathrm{t}}_{\mathrm{up}}}_{\mathrm{ev}}-\#\mathrm{ca}{{\mathrm{t}}_{\mathrm{down}}}_{\mathrm{ev}}}{n_1}-\frac{\#\mathrm{ca}{{\mathrm{t}}_{\mathrm{up}}}_{\mathrm{ne}}-\#\mathrm{ca}{{\mathrm{t}}_{\mathrm{down}}}_{\mathrm{ne}}}{n_0} $$

The NRI conditions on event status, while the RC statistic conditions on the specific cells. NRI penalizes events that move down and non-events that move up while the RC statistic penalizes individual cells that have poor fit. The two statistics were created for different purposes and cannot be formally compared: the RC statistic assesses model calibration in defined risk strata, and NRI is solely a measure of discrimination ability of one model versus the other [24–27]. From this point of view, the RC statistic is closer to another measure of goodness-of-fit—the Hosmer-Lemeshow statistic.

#### The RC statistic and the HL gof test

The Hosmer-Lemeshow test combines data across categories of predicted probabilities (often deciles). Therefore, the HL statistic can be viewed as a test of the horizontal margin of the reclassification table, had we used clinical risk categories rather than deciles as a grouping variable (Table 4). The RC statistic tests whether the fit is good in a more informed set of categories than the Hosmer-Lemeshow test statistics, which are determined by risk strata of the alternative model.

**Table 4:** Building blocks of HL and RC statistics

		Full model		
		0–5%	5–7.5%	7.5%+
Reduced model	0–5%	${\left[{KM}_{11}(t)-{\overline{p(t)}}_{11}\right]}^2$	${\left[{KM}_{12}(t)-{\overline{p(t)}}_{12}\right]}^2$	${\left[{KM}_{13}(t)-{\overline{p(t)}}_{13}\right]}^2$
	5–7.5%	${\left[{KM}_{21}(t)-{\overline{p(t)}}_{21}\right]}^2$	${\left[{KM}_{22}(t)-{\overline{p(t)}}_{22}\right]}^2$	${\left[{KM}_{23}(t)-{\overline{p(t)}}_{23}\right]}^2$
	7.5%+	${\left[{KM}_{31}(t)-{\overline{p(t)}}_{31}\right]}^2$	${\left[{KM}_{32}(t)-{\overline{p(t)}}_{32}\right]}^2$	${\left[{KM}_{33}(t)-{\overline{p(t)}}_{33}\right]}^2$
		Components of HL statistic		
		${\left[{KM}_1(t)-{\overline{p(t)}}_1\right]}^2$	${\left[{KM}_2(t)-{\overline{p(t)}}_2\right]}^2$	${\left[{KM}_3(t)-{\overline{p(t)}}_3\right]}^2$

${\left[{KM}_{31}(t)-{\overline{p(t)}}_{31}\right]}^2$ is one of the terms in the RC statistics formula. It corresponds to observations that moved from risk category 3 according to the reduced model to the risk category 1 of the full model. The reclassification table is more informative when evaluating two models because it displays the transitions from one category to another under different models

In Figs. 3, 4, and 5, we calculated the power of the Hosmer-Lemeshow test when omitting an important new biomarker, a squared term, and an interaction term to compare the power of the RC test based on clinical categories defined by 5% and 7.5% thresholds to the HL test based on deciles of predicted probabilities. We present results for an increasing baseline hazard only; simulations with decreasing baseline hazard are comparable and are included in Additional file 2: Figure S2.

From Fig. 5, the HL test is unable to detect an important omitted predictor variable for any considered sample size whereas the reclassification table does have power to detect it. In the reclassification table, information about the omitted variable is present in the form of the horizontal grouping, while for the HL statistic, this information is not provided. The lack of power of the HL statistic to detect an omitted predictor has been previously reported [13].

The RC-GND and HL tests have similar power to detect an omitted squared term (Fig. 4) when its hazard ratio is moderate to strong. The RC test also has more power to detect the omitted interaction term (Fig. 5).

The RC-GB test has more power in the considered scenarios. The GB test is semi-parametric which allows it to gain power but limits its application to the Cox proportional hazards model. The RC-GND test is non-parametric and can be applied in a wider range of scenarios. When detecting an omitted predictor variable, RC-GND and RC-GB require a sufficiently large sample size (at least 5K for an event rate of 0.1) and a large hazard ratio (2.0 and above). For smaller sample sizes, counts in the off-diagonal cells of the RC table are too small and are comparable to what could be observed under the null due to stochastic variation. Only when the signal is strong enough can it become visible over the background noise.

### Application: the Women’s Health Study

We used data from the Women’s Health Study (WHS) to illustrate how to apply the RC test in a real data example. To calculate the 10-year risk of major CVD, we used Cox proportional hazards regression with age, total cholesterol, high-density lipoprotein cholesterol (HDL), systolic blood pressure, current smoking, and diabetes as predictor variables in the full model. “Hard” CVD is defined as non-fatal myocardial infarction, a non-fatal stroke, or death from cardiovascular causes. The analysis was performed using SAS software [28] using macros available at ncook.bwh.harvard.edu. We used RC table cutoffs of 5 and 20% in this example. In Table 5, we tested seven reclassification tables, comparing the full model to one without the predictor in the first column of Table 5 (reduced model).

**Table 5:** Results of seven RC statistics tests, comparing the full model to one without the predictor in the first column of this table (reduced model)

			Based on pp from the reduced model		Based on pp from the full model	
	Beta	*p*-value (beta)	RC statistic	*p*-value	RC statistic	*p*-value
AGE	5.08	< 0.001	80.49	< 0.001	3.97	0.86
TOTC	0.93	< 0.001	7.48	0.28	4.16	0.65
HDLC	− 0.95	< 0.001	8.00	0.24	6.71	0.35
CURRSMOKING	1.01	< 0.001	24.84	< 0.001	7.38	0.39
SBP	3.90	< 0.001	57.04	< 0.001	8.60	0.28
DIABETES	1.32	< 0.001	33.66	< 0.001	5.74	0.57
RANDOM	− 0.05	0.20	2.99	0.56	3.08	0.54

The GND test was used for testing the reclassification table. We used age, total cholesterol (TOTC), HDL cholesterol (HDLC), current smoking (CURRSMOKING), systolic blood pressure (SBP), and diabetes status (DIABETES) as well as a random null variable (RANDOM) as predictor variables in the full model

In Table 5, the beta coefficients are significant for all six informative predictor variables. However, total cholesterol and HDL cholesterol have a non-significant effect on reclassification into clinical categories: corresponding *p*-values when the reduced model probabilities were used show a good fit (*χ*^2^ = 7.48 and 8.00, one-sided *p*-values = 0.28 and 0.24), while the RC statistic using the new model is also not significant. In that case, we would choose the more parsimonious model without the variable in question. This finding is due to the fact that total cholesterol and HDL cholesterol are correlated and result in very few clinical reclassifications. It also illustrates our point that a significant biomarker with a small beta estimate can result in a limited number of reclassifications, and therefore, it will have only minor impact in clinical practice. In contrast, a removal of current smoking from the full model results in a highly significant RC-GND test when predicted probabilities were used from the reduced model (*χ*^2^ = 24.84, *p*-value < .001).

When the predicted probabilities were used from the model with smoking, a good fit was found (*χ*^2^ = 7.38, *p*-value = 0.39), confirming that the full model reclassifies observations into better calibrated groups, using Kaplan-Meier to estimate the observed event rate in each group.

In the last row, we added an uninformative biomarker to the full model. We expected the RC-GND test to be non-significant no matter whether one uses the full or reduced model to calculate predicted probabilities in a cell. Indeed, both tests had non-significant *p*-values (.56 and .54), indicating that the smaller model has a good fit and the addition of the new biomarker does not improve it. Nor does it negatively affect it (because the full model with uninformative biomarker is also well calibrated). However, we prefer a more parsimonious model since it performs at least as well. In practice, if the uninformative marker displayed no association with the outcome using likelihood ratio testing or other established methods, we would not proceed to examine reclassification.

## Discussion

Risk reclassification extends evaluation of risk prediction models from traditional approaches informed by discrimination and calibration measures (such as the AUC and Hosmer-Lemeshow test) toward assessments focused on the clinical relevance of a new model and implications on present-day treatment decisions [11, 29–31]. Appropriate statistical methodology for measures of reclassification is still an active field of research, and it is crucial to develop valid statistical tests [11].

The RC statistic is an important reclassification tool which compares performance of predictive models with respect to clinically relevant decision categories [12, 15, 32–34]. Performance of new markers may vary across subgroups [35], and it will be of interest to identify subgroups for which the new markers may or may not be useful. The reclassification table helps to visualize and to better understand movements between categories, see which groups of patients are influenced more by the inclusion of a given biomarker, and test significance of improvement.

The RC test falls between the moderate and strong calibration categories in the Van Calster hierarchy of risk calibration [4]. It goes beyond testing in standard Hosmer-Lemeshow risk groups defined by a single model and looks at movements across risk groups defined by both full and reduced models. It also can be repeated for a variety of covariate patters but does not exhaust all possibilities. Therefore, it is not performing a full assessment to assure “strong” calibration, but it goes beyond the moderate calibration within standard HL deciles.

In this paper, we extend the RC statistic to the survival setting with higher censoring rates. We recommend using the RC-GND test to test the reclassification table with survival data. The RC-GND test is fully non-parametric and therefore can be applied in a wide variety of situations. It does not refit the baseline hazard as, for example, the Grønnesby-Borgan test does [19], so it can detect a lack of calibration in either model.

In our simulations, the RC-GND is very sensitive to omission of an important predictor variable (Fig. 3), a quality that some other goodness-of-fit tests do not share. It achieves 80% power when an important new predictor with HR > 2.0 was omitted, though this depends on the sample size. Many authors noted that improving discrimination of a strong baseline model also requires a strong enough predictor variable [36]. Therefore, if an established model has a relatively strong discrimination (as for example Framingham ATPIII model with c-statistic of 0.83 for women [37]), then to improve significantly in terms of discrimination (measured by c-statistic) or in terms of calibration, a strong predictor variable is required.

Limitations of the RC statistic include its dependence on the existence of clinically relevant risk stratification categories. Oftentimes, however, clinically relevant cutoffs are not established. In this situation, we recommend producing an RC table for a set of sensible risk cut points, possibly centered around the disease incidence [13]. As we have mentioned earlier, treatment guidelines in several fields do rely on established risk categories [1, 9]. In this situation, another important issue is how sharp are the boundaries of clinically established risk categories. If a patients’ risk falls in a proximity of a cutoff point (for example risk of 7.4% with the cutoff of 7.5%), then how certain are we that the treatment regimen should be that for intermediate risk rather than for a high risk? It may make sense to establish “transition areas” where assignment to a risk category is mute. A prediction confidence interval for the predicted risk is available in most statistical software packages and can be included in risk calculators for patient’s estimated risk. If prediction confidence interval covers the threshold, then patient’s risk falls in the transition area from one risk category to another. This is an important information to consider when making a treatment decision. Alternatively, if there is a single risk cutoff, then additional cutoffs on either side of it could be established in a four-category classification to allow for uncertainty. A single category below the cut point could also be used for “watchful waiting” or further follow-up.

Additionally, we also did not consider competing events, although these could be taken into account in a similar fashion [38]. Sensitivity to small cell sizes is another disadvantage of the RC-GND test. If sample size is too small and the hazard ratio of the new biomarker is not large enough, the RC-GND test does not have enough power to detect an improvement over the baseline model, and therefore, the RC-GND test is too conservative.

We compared the Hosmer-Lemeshow style test to the RC test. The Hosmer-Lemeshow test can be viewed as a test of the margin of the reclassification table. An important limitation of the HL test is its inability to detect an omitted biomarker. Our Fig. 3 illustrates that non-significance of the HL test should not be viewed as an evidence that the model contains all important biomarkers. If a decision must be made about inclusion of a biomarker in a risk-prediction model, the HL statistic will always show a good fit if the categories are defined by a model without that biomarker. In other words, if a model has a good fit based on the HL test, it does not guarantee at all that the model has all important variables in it. In the reclassification table, the biomarker is used to define risk categories, so the RC statistic is sensitive to the omission of an important biomarker. In general, however, these measures focus on calibration, and more direct model comparisons, such as likelihood ratio or related measures, can be used to assess whether a new biomarker is important.

## Conclusions

The reclassification table is a step toward better understanding of the clinical utility of one model versus the other. It can be used to visualize movements of patients across categories and examine whether a new model has an impact on clinical treatment assignment. The associated RC statistics can assess calibration of both models and indicate areas where fit may be lacking. Unlike the GB test, the GND test does not rely on the assumptions of proportionality of hazards [19]; therefore, we recommend the GND test for inference in a variety of settings, particularly when the Cox model is not in use.

## Additional files

Additional file 1: **Figure S1.** Size of the RC-GND test (3) and RC-GB (score test). Comparing full (*y*~ *x*_1_ + *x*_2_) and reduced (*y*~ *x*_1_) models with decreasing (top row) and increasing (bottom row) baseline hazard functions. *N* = 5000, *p* = 0.1, collapse when *ev*_*g*_ < 5. (PDF 59 kb)

Additional file 2: **Figure S2.** Power of RC-GND and RC-GB for a decreasing baseline hazard. Summary of the simulations is presented in the Supplementary **Table S1.** An important predictor variable was omitted (the top row), a squared term was omitted (the middle row), and an interaction term was omitted (the bottom row). Event times follow Weibull distribution with a decreasing baseline hazard as discussed in the section “Simulations setup” and the Supplementary **Table S1.** for the sample size of 5000, event rate of 0.1, cells were collapsed when number of events in a cell was less than five. (PDF 48 kb)

Additional file 3: **Table S1.** Outline of simulations used to generate Fig. 1. (DOCX 24 kb)

## Abbreviations

AUC: Area under the Receiver Operating Characteristic Curve
CVD: Cardiovascular disease
GB test: Grønnesby-Borgan test
GND test: Greenwood-Nam-D’Agostino test
gof: Goodness-of-fit
HL test: Hosmer-Lemeshow goodness-of-fit test
NRI: Net Reclassification Improvement
RC statistic: Reclassification Calibration statistic
WHS: Women’s Health Study
