# Supplementary material for: Reclassification calibration test for censored survival data: performance and comparison to goodness-of-fit criteria
Source: Diagn Progn Res. 2018 Jul 26;2:16. doi: 10.1186/s41512-018-0034-5 (PMC6456068; doi:10.1186/s41512-018-0034-5)
Supplement: Supplementary file 1 — Figure S1. Size of the RC-GND test (3) and RC-GB (score test). Comparing full (y~ x1 + x2) and reduced (y~ x1) models with decreasing (top row) and increasing (bottom row) baseline hazard functions. N = 5000, p = 0.1, collapse when evg < 5. (PDF 59 kb) [file 41512_2018_34_MOESM1_ESM.pdf]

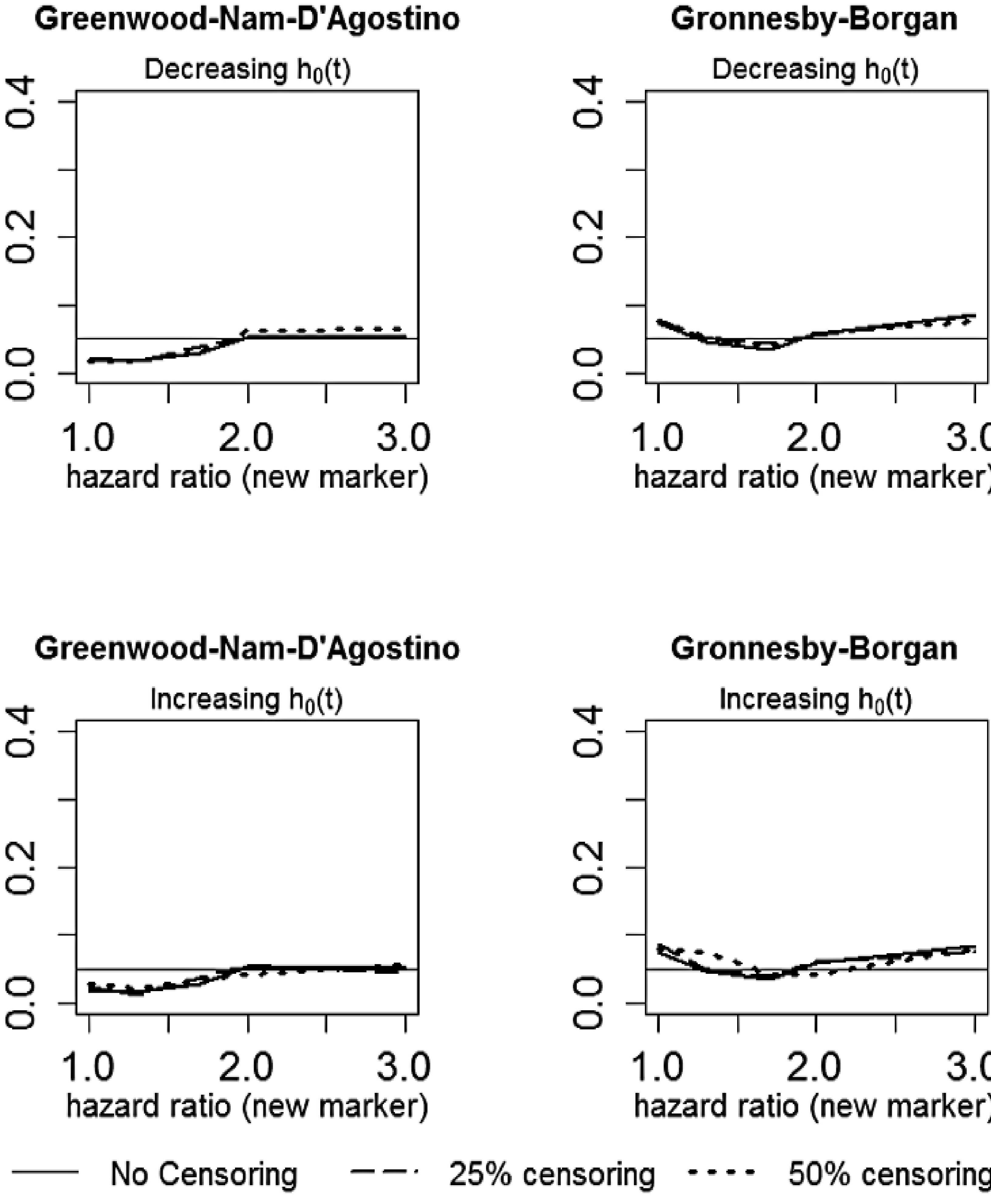

Greenwood-Nam-D'Agostino
Decreasing $h_0(t)$
Gronnesby-Borgan
Decreasing $h_0(t)$
Greenwood-Nam-D'Agostino
Increasing $h_0(t)$
Gronnesby-Borgan
Increasing $h_0(t)$
0.0
0.2
0.4
1.0
2.0
3.0
hazard ratio (new marker)
No Censoring
25% censoring
50% censoring
