# Supplementary figures and images for: Reclassification calibration test for censored survival data: performance and comparison to goodness-of-fit criteria
Source: Diagn Progn Res. 2018 Jul 26;2:16. doi: 10.1186/s41512-018-0034-5 (PMC6456068; doi:10.1186/s41512-018-0034-5)

**RC-GND**

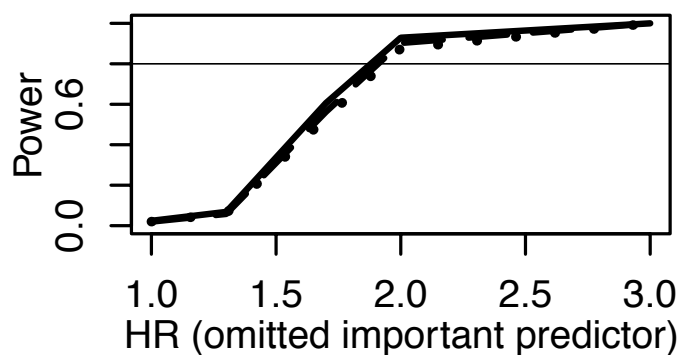

**RC-GB**

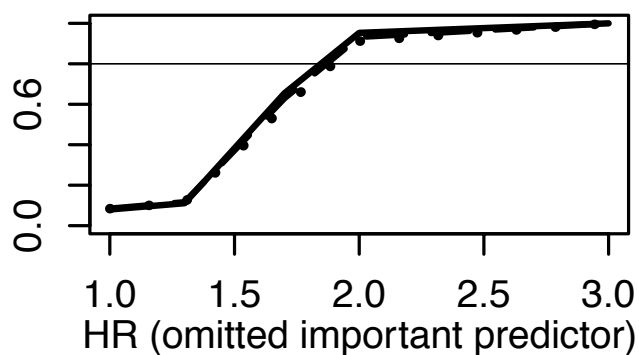

**RC-GND**

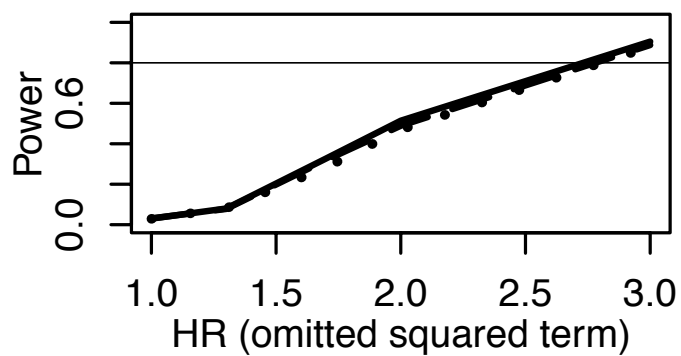

**RC-GB**

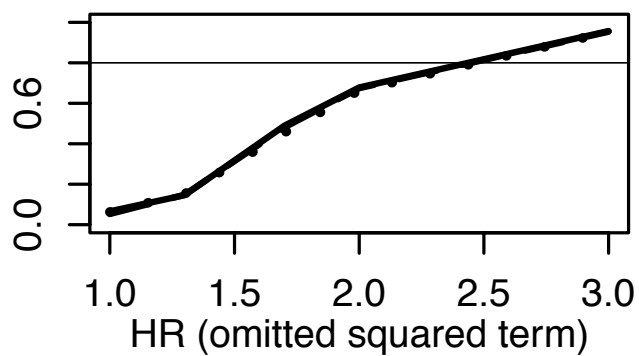

**RC-GND**

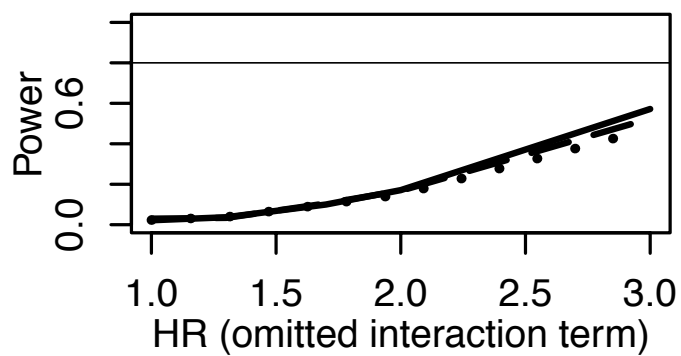

**RC-GB**

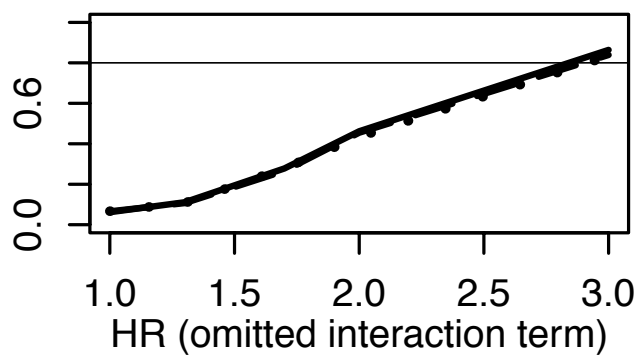

— No Censoring    - - - 25% censoring    . . . 50% censoring

Supplement: Supplementary file 2 — Figure S2. Power of RC-GND and RC-GB for a decreasing baseline hazard. Summary of the simulations is presented in the Supplementary Table S1. An important predictor variable was omitted (the top row), a squared term was omitted (the middle row), and an interaction term was omitted (the bottom row). Event times follow Weibull distribution with a decreasing baseline hazard as discussed in the section “Simulations setup” and the Supplementary Table S1. for the sample size of 5000, event rate of 0.1, cells were collapsed when number of events in a cell was less than five. (PDF 48 kb) [file 41512_2018_34_MOESM2_ESM.pdf]
