# Supplementary material for: Reclassification calibration test for censored survival data: performance and comparison to goodness-of-fit criteria
Source: Diagn Progn Res. 2018 Jul 26;2:16. doi: 10.1186/s41512-018-0034-5 (PMC6456068; doi:10.1186/s41512-018-0034-5)
Supplement: Supplementary file 3 — Table S1. Outline of simulations used to generate Fig. 1. (DOCX 24 kb) [file 41512_2018_34_MOESM3_ESM.docx]

**Additional file 3**

**Outline of Simulations Used to Generate Figure 1.**

Event times were generated from the Weibull distribution with the shape parameter$\alpha$ set to 3.0 for models with increasing baseline hazard and 0.3 for models with the decreasing baseline hazard. Scale parameter of the Weibull distribution was proportional to exponentiated risk score of the data-generating model, i.e. rs=ln(8)x_1_ +ln(1.0,1.3,1.7,2.0,3.0)x_2_, where *x_1_~*N(0,0.5) and *x_2_~*N(0,0.5). Scale parameter of the Weibull distribution was also calibrated to have 0.1 event incidence rate. Censoring times were uniformly distributed to generate 0%, 25% and 50% censoring rates. This data was fit with Cox proportional hazards regression model with $S_{full}\left( t \right)=\exp\left( -t^{\alpha}\lambda\exp\left( \beta_{1}x_{1}+\beta_{2}x_{2} \right) \right); S_{redu}\left( t \right)=exp(-t^{\alpha}\lambda exp(\beta_{1}x_{1}))$. The column-wise and row-wise categories in the RC table were formed using the reduced model (columns) and full model (rows) with cutoffs of 5% and 20%. To calculate size, expected counts in the RC were calculated under the data-generating model.

**Table S1. Summary of Simulations Used to Generate Figures 3-5.**

| Risk score of the data-generating model^‡^ | Risk score of the reduced model | Distribution of predictor variables | Censoring rates | Sample size |
| --- | --- | --- | --- | --- |
| *when omitting an important predictor*  ln(8)x_1_ +ln(1.0,1.3,1.7,2.0,3.0)x_2_ | ln(8)x_1_ | x_1_~N(0, 0.5), x_2_~N(0, 0.5) | 0%, 25%, 50% | 1K, 5K, 10K |
| *when omitting a squared term*  ln(8)x_1_ +ln(1.0,1.3,1.7,2.0,3.0)x_1_^2^ | ln(8)x_1_ | x_1_~N(0, 0.5) | 0%, 25%, 50% | 1K, 5K, 10K |
| *when omitting an interaction term*  ln(8)*x_1_ + ln(8)x_2_ +ln(1.0,1.3,1.7,2.0,3.0)x_1_ x_2_ | ln(8)x_1_ + ln(8)x_2_ | x_1_~N(0, 0.5), x_2_~Bin(1, 0.116^#^) | 0%, 25%, 50% | 1K, 5K, 10K |

^‡^We used baseline hazards within the ranges of the Cox model coefficients in original ATPIII model ([1](#_ENREF_1)).

^#^When generating a binary predictor variable, we used the prevalence of current smoking in WHS data at baseline (0.116) as a parameter of the binary distribution.

**The Grønnesby-Borgan test.**

Using martingale theory Grønnesby and Borgan developed a test of fit for Cox proportional hazards regression models ([2](#_ENREF_2)). It is based on the difference between the observed and expected number of events in deciles but it can be applied to any grouping. This difference in group *g* at time *t* is a sum of martingale residuals and is denoted as $M_{i}\left( t \right)$:

This difference in group *g* at time *t* is a sum of martingale residuals and is denoted as $M_{i}\left( t \right)$:

$H_{g}\left( t \right)=\sum_{i\in g} M_{i}\left( t \right)=\sum_{i\in g} N_{i}\left( t \right)-\sum_{i\in g} \int_{0}^{t} h_{0}\left( s \right)\exp\left( \boldsymbol{x}_{\boldsymbol{i}}^{\boldsymbol{T}}\boldsymbol{\beta} \right)Y_{i}\left( s \right)\mathrm{ds}$, (A1)

where *g* is a group, *N_i_(t)* is an event indicator for person i by time t, $Y_{i}(t)$ is at-risk indicator for person *i* and $\sum_{i\in g} \int_{0}^{t} h_{0}\left( s \right)\exp\left( \boldsymbol{x}_{\boldsymbol{i}}^{\boldsymbol{T}}\boldsymbol{\beta} \right)Y_{i}\left( s \right)\mathrm{ds}$ is the usual expected number of events in group *g* at time *t* using a counting process formulation([3](#_ENREF_3)). $M_{i}\left( t \right)$ in formula (3) is a martingale residual at time t. When used with estimates of $\boldsymbol{\beta}$ in $M_{i}(t)$, it becomes approximate martingale residual $\hat{M}_{i}(t)$. $\hat{H_{1}\left( t \right),\ldots,H_{G-1}\left( t \right)}$ is a vector of sums of approximate martingale residuals has a multivariate Gaussian distribution, with variance-covariance matrix $\hat{\Sigma}$ calculated in([2](#_ENREF_2)), with

$\chi_{GB}^{2}\left( t \right)=\left( \hat{H}_{1}\left( t \right),\ldots,\hat{H}_{G-1}\left( t \right) \right)\hat{\Sigma}^{-1}(t)\left( \hat{H}_{1}\left( t \right),\ldots,\hat{H}_{G-1}\left( t \right) \right)^{T}\sim\chi_{G-1}^{2}$. (A2)

May and Hosmer showed([4](#_ENREF_4)) that this test is algebraically equivalent to a score test, which is implemented in most statistical software packages. When proportionality of hazards assumption holds we can apply the GB test (i.e. score test) to the RC table. Therefore instead of calculating (A2) we can perform the score test: add indicator variables for each cross-classified cell to the model of interest, simultaneously forcing their coefficients to be set to zero and coefficients of other variables to remain the same. Under the null the derivative of the likelihood for this set of parameters is zero.

1. Expert Panel on Detection E. Executive summary of the Third Report of the National Cholesterol Education Program (NCEP) expert panel on detection, evaluation, and treatment of high blood cholesterol in adults (Adult Treatment Panel III). Jama. 2001;285(19):2486.

2. Grønnesby JK, Borgan Ø. A method for checking regression models in survival analysis based on the risk score. Lifetime data analysis. 1996;2(4):315-28.

3. Andersen PK, Borgan O, Gill RD, Keiding N. Statistical models based on counting processes: Springer Science & Business Media; 2012.

4. May S, Hosmer DW. A simplified method of calculating an overall goodness-of-fit test for the Cox proportional hazards model. Lifetime data analysis. 1998;4(2):109-20.
